# Assessment of atropine-sufentanil-atracurium anaesthesia for endotracheal intubation: an observational study in very premature infants

**DOI:** 10.1186/1471-2431-14-120

**Published:** 2014-05-07

**Authors:** Xavier Durrmeyer, Sonia Dahan, Pierre Delorme, Sabine Blary, Gilles Dassieu, Laurence Caeymaex, Ricardo Carbajal

**Affiliations:** 1 Epidemiology and Biostatistics Centre, Obstetrical, Perinatal and Pediatric Epidemiology Team, Université Pierre et Marie Curie Paris VI, Paris, Inserm UMRS 1153, France; 2 NICU, Centre Hospitalier Intercommunal, Créteil, France; 3 CRC, Centre Hospitalier Intercommunal, Créteil, France; 4 Paediatric Anaesthesia Department, Centre Hospitalier Intercommunal, Créteil, France; 5 Service des Urgences Pédiatriques, Hôpital Trousseau, AP-HP, Paris, France

**Keywords:** Endotracheal intubation, Newborn, Opioids, Muscle relaxant, Pain, Hypercapnia

## Abstract

**Background:**

Premedication before neonatal intubation is heterogeneous and contentious. The combination of a short acting, rapid onset opioid with a muscle relaxant is considered suitable by many experts. The purpose of this study was to describe the tolerance and conditions of intubation following anaesthesia with atropine, sufentanil and atracurium in very premature infants.

**Methods:**

Monocentric, prospective observational study in premature infants born before 32 weeks of gestational age, hospitalised in the NICU and requiring semi-urgent or elective intubation. Intubation conditions, heart rate, pulse oxymetry (SpO_2_), arterial blood pressure and transcutaneous PCO_2_ (TcPCO_2_) were collected in real time during 30 minutes following the first drug injection. Repeated physiological measurements were analysed using mixed linear models.

**Results:**

Thirty five intubations were performed in 24 infants with a median post conceptional age of 27.6 weeks and a median weight of 850 g at the time of intubation. The first attempt was successful in 74% and was similar for junior (75%) and senior (74%) operators. The operator rated conditions as “excellent” or “good” in 94% of intubations. A persistent increase in TcPCO_2_ as compared to baseline was observed whereas other vital parameters showed no significant variations 5, 10, 15 and 30 minutes after the first drug injection. Eighteen (51%) desaturations (SpO2 less than or equal to 80% for more than 60 seconds) and 2 (6%) bradycardia (heart rate less than100 bpm for more than 60 seconds) were observed.

**Conclusion:**

This drug combination offers satisfactory success rate for first attempt and intubation conditions for the operator without any significant change in heart rate and blood pressure for the patient. However it is associated with frequent desaturations and a possible persistent hypercapnia. SpO_2_ and PCO_2_ can be significantly modified during neonatal intubation and should be cautiously followed in this high-risk population.

## Background

Endotracheal intubation is a frequent procedure in the Neonatal Intensive Care Unit (NICU). Although neonatal awake intubation is not recommended, except for emergency situations, no consensus exists to date supporting the use of a drug or a drug combination in neonates [1]. Clinical studies addressing premedication before endotracheal intubation in neonates include randomised, controlled trials vs placebo [2,3] or comparison of different regimens [4-7] and observational studies [8-12]. Based on these, experts have recommended the use of a short onset opioid [1,13,14]. Fentanyl is the most studied synthetic opioid in this context [6,8,9,11]. In contrast with the scarce data regarding sufentanil use in neonates [15] and the latest expert recommendations [1], French neonatologists use sufentanil more frequently than fentanyl for intubating neonates either in the NICU [16] or in the delivery room [17]. An increase in sufentanil use in German NICUs has also been recently reported [18]. Some trials have demonstrated the interest of associating a short onset opioid with a muscle relaxant [4,6,11,19]. However muscle relaxants are rarely used in France [16,17,20]. Randomised controlled trials (RCT) usually provide reliable evidence. However, real practice does not always correspond to bedside conditions [16]. As such, observational studies are frequently beneficial in providing data from actual clinical practice [21,22]. The latter studies are necessary to objectively evaluate the consequences of actual practices. In February 2007, we implemented and prospectively evaluated a protocol in our NICU for anaesthesia before endotracheal intubation in non-life threatening situations combining atropine, sufentanil and atracurium. This protocol was elaborated in collaboration with the paediatric anaesthesiologists from our institution and written after a review of the existing published evidence at that time. Our objectives were to describe the real conditions of the procedure, to document tolerance and to collect adverse events in a group of premature infants born under 32 weeks of gestational age (GA).

## Methods

### Anaesthesia protocol

Infants received intravenously 15 μg/kg of atropine as a bolus followed by 0.2 μg/kg of sufentanil over 60 seconds and 0.3 mg/kg of atracurium over 30 seconds. If paralysis was not obtained within 2 minutes after atracurium injection, an additional dose of 0.1 mg/kg could be given and repeated once (maximal atracurium cumulative dose 0.5 mg/kg).

### Intubation procedure

In non intubated patients, a bag-valve-mask ventilation (Ambu© Mark IV baby, Denmark), without positive expiratory pressure, was performed from the beginning of atropine injection. In intubated patients who needed an endotracheal tube (ETT) change, the ETT was removed once the new ETT had been introduced in the opposite nostril and the laryngoscope was inserted. FiO_2_ was set at the discretion of the operator and no target SpO_2_ nor minimal duration of preoxygenation was recommended due to uncertainty on optimal preoxygenation in this population [23]. When a junior operator performed the procedure, a senior operator was always present to take over the procedure in case of failure. A maximum of two attempts were allowed for junior operators. Intubation in our unit was always nasotracheal and performed using an appropriate sized Macgill forceps.

### Data collection

This observational study was conducted in a single level 3 unit between February and August 2007. Data were collected prospectively on a standardised form by an observer who was not involved in the procedure (resident, nurse, fellow or consultant). Patients’ clinical characteristics were collected from his/her chart and included gestational age at birth, birth weight, sex, postnatal age, corrected age and weight at the time of intubation, main indication for intubation and opioid or benzodiazepine administration within the 24 hours prior to the intubation.

#### Intubation procedure

The duration of the procedure was defined as the time between first laryngoscope insertion and definitive tube fixation to the nose with a tape. Each laryngoscope insertion was considered as an attempt. Fellows and consultants were considered as senior operators and residents as junior operators. Once intubated, infants were ventilated with a pressure-limited ventilator (Babylog 8000, Dräger, Lubeck, Germany). Ventilator settings were collected 1 minute before the first drug injection (baseline = M-1), then at 5, 10, 15 and 30 minutes after first drug injection (referred as M5, M10, M5 and M30).

#### Vital signs collection

Heart rate, pulse oxymetry and non invasive blood pressure (Viridia, Philips Medical Systems, Andover, MA) were continuously monitored and values were collected 1 minute before the first drug injection (baseline = M-1), then at 5, 10, 15 and 30 minutes after first drug injection (referred as M5, M10, M5 and M30). The lowest heart rate and pulse oxymetry values during the intubation procedure were collected. Whenever possible and available, transcutaneous CO_2_ partial pressure (TcPCO_2_) (Intellivue TcG10, Philips Medical Systems, Andover, MA) was continuously recorded and collected at the previously specified time points.

#### Quality of sedation

Data regarding the quality of intubation conditions were collected immediately after completion of the procedure by the operator who succeeded the intubation according to the following scale adapted from Hans [24] and Cooper [25]:

– Excellent: Relaxed jaw and open vocal cords and no movement when inserting ETT

– Good: Relaxed jaw and open vocal cords and mild movements when inserting ETT

– Acceptable: Mild jaw contraction and/or moving vocal cords and/or cough when inserting ETT

– Poor: Jaw contraction or closed vocal cords or intense cough or rigidity when inserting ETT.

#### Adverse events

Expected adverse events included:

– Thoracic rigidity diagnosed by the operator without any specific predefined criteria.

– Desaturation arbitrarily defined as a SpO_2_ value ≤ 80% for more than 60 consecutive seconds. Duration was measured with a stopwatch.

– Bradycardia defined as heart rate < 100 bpm lasting more than 60 seconds. This was obtained from a posteriori monitoring recordings analysis.

– Upper airway injury defined as presence of blood in the mouth during or after the procedure.

Any other adverse events could be recorded freely on the data collection form.

### Statistical analysis

#### Data reporting

Descriptive statistics were expressed as median or mean according to their distribution. Intubation conditions and incidence of desaturations were compared between junior and senior operators using Fisher’s exact test or Mann–Whitney U test.

#### Predictive model

We used mixed linear models in order to predict the changes in heart rate, pulse oxymetry, mean arterial blood pressure (MAP) and TcPCO2 over time based on our observations. Changes in the parameters were modeled using fractional polynomials in order to take into account non-linear time trends. In order to ensure independent observations, only the first episode of intubation was considered for the building of these models if multiple intubations were performed in the same infant at different time points.

A p value below 0.05 was considered significant. All analyses were performed using Stata v11.2 software (Statacorp, Texas, USA).

### Ethics

No other consent than consent to usual standard care from the parents was requested since the implementation of the protocol was part of a standard of care modification process. The local ethics committee (groupe de reflexion éthique de l’Hôpital Intercommunal de Créteil) approved the anonymous collection of data and their publication.

## Results

### Population

Between February and August 2007, we collected data on 35 intubations in 24 infants born under 32 weeks of gestational age. Studied infants (13 boys, 11 girls) had a median (range) gestational age at birth and birth weight of 26.0 (23.9-31.6) weeks and 850 (480–1860) g, respectively. During the study period 17 infants underwent one intubation, 3 infants 2 intubations and 4 infants 3 intubations.

### Intubation conditions

All studied intubations were semi-urgent or planned intubations. Their conditions are summarized in Table 1. First attempt was successful in 74% and conditions were considered “excellent” or “good” by operators in 94% of intubations according to our 4-level quality of sedation assessment. One infant required an additional 0.1 mg/kg dose of atracurium (cumulated dose 0.4 mg/kg) and 3 infants required 2 additional 0.1 mg/kg doses of atracurium (cumulated dose 0.5 mg/kg). No drug or dosing error was observed. Mean +/- SE (time points) delta pressures (i.e. peak inspiratory pressure - PEEP) for infants who were receiving invasive ventilation were 14.0 +/- 3.2 (M5), 13.2+/- 2.3 (M10), 13.3 +/- 2.7 (M15) and 13.1 +/- 2.4 (M30) cm H_2_O. Mean +/- SE (time points) set respiratory rates were 68.7 +/- 9.8 (M5), 67.3 +/- 9.7 (M10), 70.9 +/- 9.4 (M15), 71.9 +/- 9.6 (M30) cycles/minute.

**Table 1:** Condition of 35 intubations in infants < 32 weeks GA

**Condition**	**Results**
Median post natal age at intubation in days [IQR]	10 [4-16]
Median post conceptional age at intubation in weeks [IQR]	27.6 [26.3-28.9]
Median weight at the time of intubation in g [IQR]	850 [740-1000]
Ventilatory support at the time of intubation, n (%)	
Invasive ventilation	10 (28.5%)
Non-invasive ventilation	23 (66%)
Spontaneous breathing	2 (5.5%)
Drug administration in the 24 hours prior to intubation, n (%)	
Benzodiazepines	6 (17%)
Opioids	5 (14%)
Indication for intubation, n (%)	
Respiratory failure	12 (34%)
Apnea	10 (28.5%)
ETT change	10 (28.5%)
Surgery	3 (9%)
Median FiO_2_ one minute before atropine injection, % [IQR]	37 [24-60]
Number of attempts, n (%)	
1	26 (74%)
2	5 (14%)
3	2 (6%)
4	1 (3%)
5	1 (3%)
Median duration of intubation^a^, s [IQR]	180 [110-328]
Quality of sedation, n (%)	
Excellent	28 (80%)
Good	5 (14%)
Acceptable	2 (6%)
Poor	0 (0%)
Desaturation <80%, > 60 seconds, n (%)	18 (51%)
Bradycardia < 100 bpm, > 60 seconds, n (%)	2 (6%)
Median lowest saturation, % [IQR]	58 [48-79]
Median lowest heart rate, bpm [IQR]	141 [120-157]

^a^Time between the first laryngoscope insertion and definitive tube fixation to the nose with a tape.

IQR: Interquartile range.

### Vital signs

**Figure 1:**
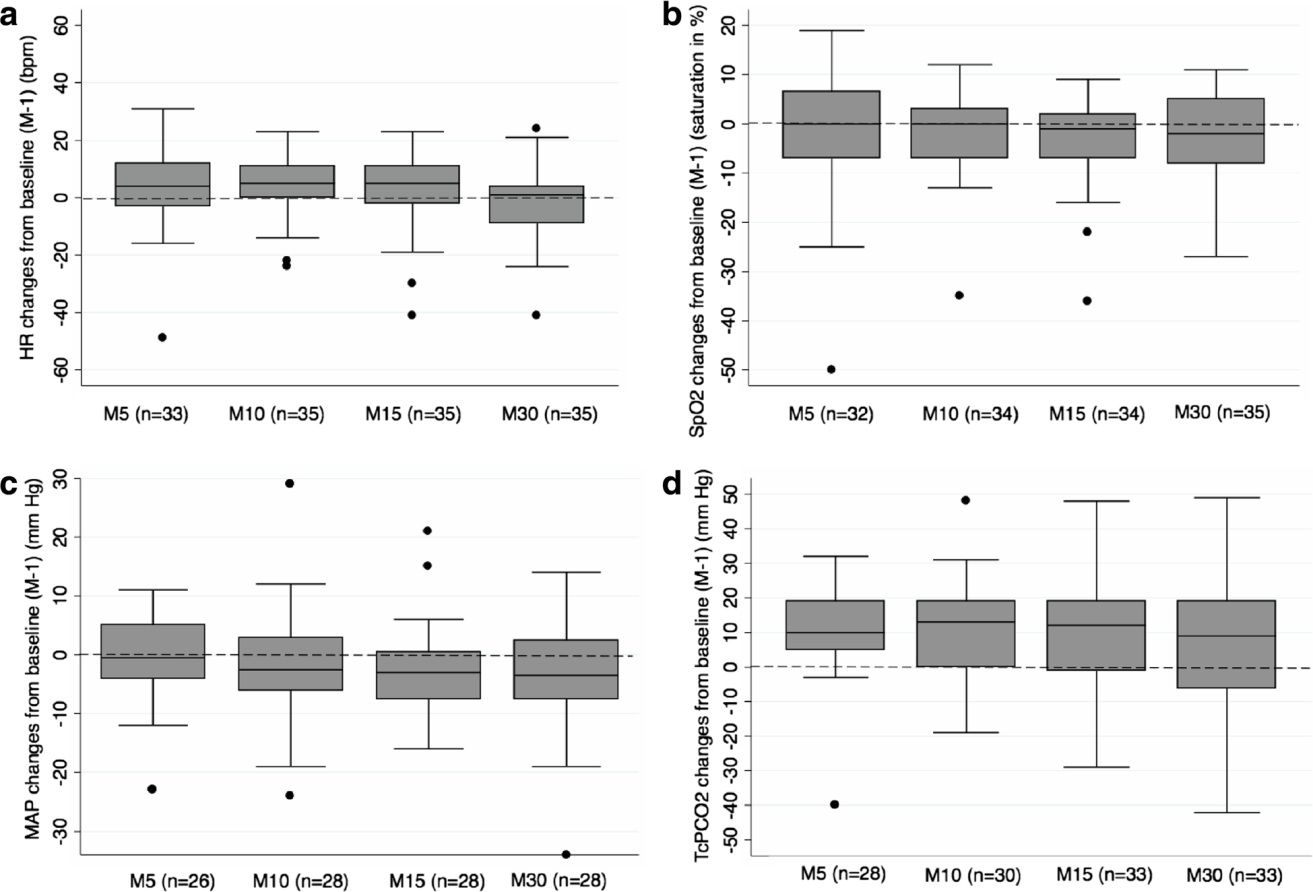
**Absolute changes from baseline (median, IQR, extremes) in heart rate (a), SpO**_**2 **_**(b), mean arterial blood pressure (c) and TcPCO**_**2 **_**(d) at observed time points.** X axis (time points): M-1: one minute before atropine injection, M5: 5 minutes after atropine injection, M10: 10 minutes after atropine injection, M15: 15 minutes after atropine injection, M30: 30 minutes after atropine injection. Y axis: Numerical difference from baseline value for each parameter. Boxes represent values between the 1^st^ and the 3^rd^ quartile. The bar inside the box denotes median value. The adjacent values are the most extreme values within 1.5 inter-quartile range of the nearer quartile. Black dots are outliers outside adjacent values. Numbers in parenthesis denote the number of available measures at each time point. HR: heart rate, MAP: mean arterial blood pressure.

Figure 1a-1d illustrate absolute changes from baseline over time in heart rate, pulse oxymetry, MAP and TcPCO_2_, respectively. Heart rate, pulse oxymetry and MAP values collected at specified time points remained relatively stable as compared to baseline (Figure 1a, 1b, 1c). TcPCO_2_ values generally increased from baseline although they tented to be more dispersed over time (Figure 1d).

### Adverse events

A desaturation ≤ 80% lasting > 60 seconds was observed in 18 intubations (51%) (Table 1). During the 35 analysed intubations, we observed 2 bradycardias < 100 bpm lasting longer than 60 seconds, 3 traumatic injuries of upper airways and 8 chest-wall rigidities. In all cases of reported chest-wall rigidity but one, a prolonged desaturation was observed. No other adverse event was reported.

### Experience of the operator

A junior and a senior operator carried out the first attempt for intubation in 12 and 23 intubations respectively. Infants’ median GA at birth, birth weight, post conceptional age at intubation, age at intubation and weight at intubation were comparable for junior and senior operators. First intubation attempts were successful for 75% and 74% of junior and senior operators respectively (p = 1.00). Median durations of intubation were 199 and 165 s. for junior and senior operators respectively (p = 0.90). The frequencies of desaturations < 80% lasting > 60 s. were 42% and 57% for junior and senior operators respectively (p = 0.49).

### Predictive model

**Figure 2:**
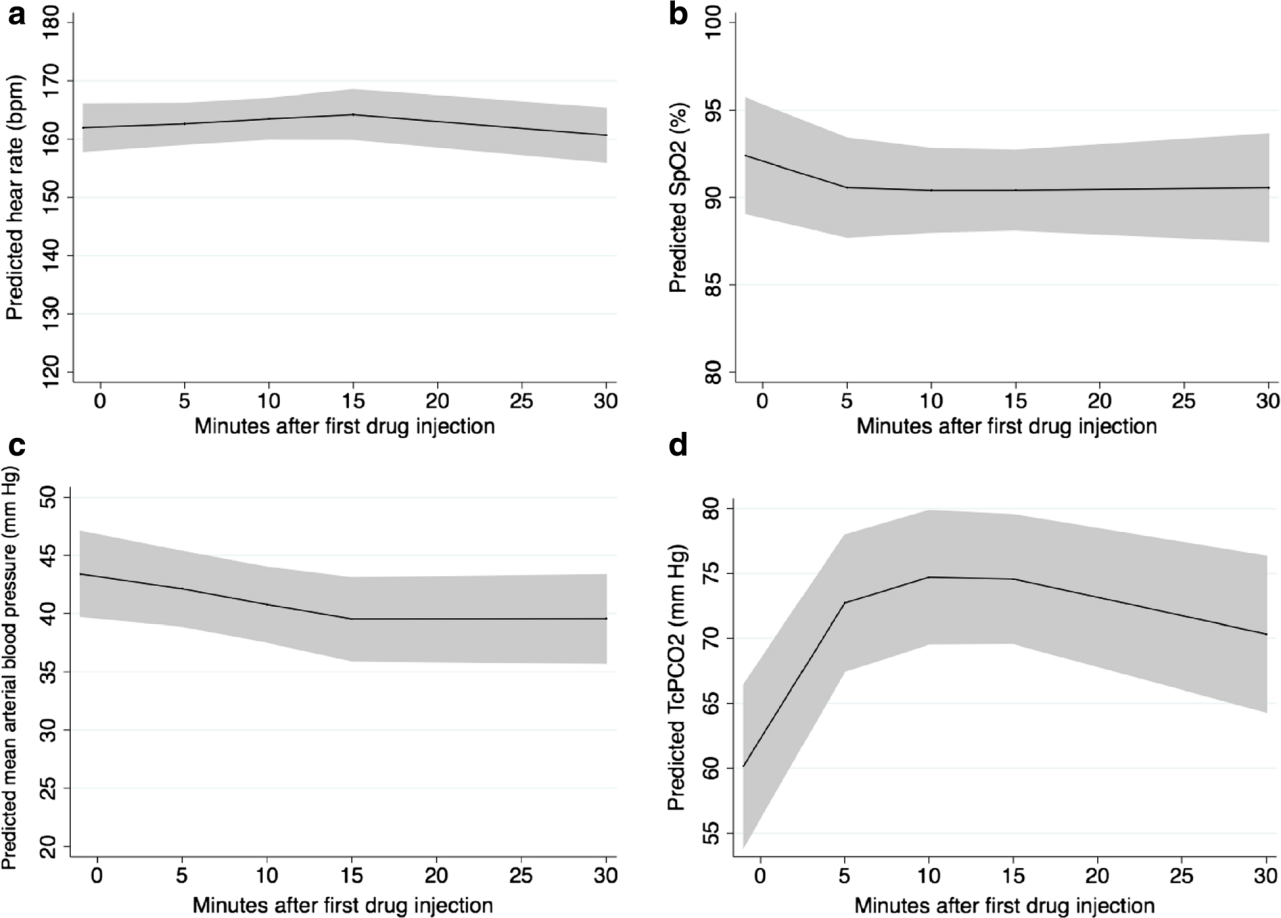
**Predictive models (mean, 95% CI) for the evolution of heart rate (a), SpO2 (b), mean arterial blood pressure (c) and TcPCO2 (d) over time.** X axis: Time after first drug injection in minutes. Y axis: Polynomial of degree 2 predictive model for heart rate **(a)**, pulse oxymetry **(b)**, mean arterial blood pressure **(c)** and TcPCO_2_**(d)** changes over time. The solid black line illustrates estimated mean values for each studied variable over time. The gray zone illustrates the 95% confidence interval for these estimated values.

Predicted heart rate, pulse oxymetry, MAP and TcPCO_2_ over time using mixed linear models are illustrated in Figure 2a-2d respectively. We found no statistically significant time trends for heart rate, pulse oxymetry and mean arterial blood pressure whereas TcPCO_2_ changed significantly over time (p < 0.001).

## Discussion

This observational study showed that in very premature infants the association of atropine, sufentanil and atracurium provided good intubation conditions as rated by the operator and a 74% success rate for first attempt. No significant changes in heart rate, mean arterial blood pressure or pulse oxymetry recorded at predefined time points were observed. Desaturations below 80% lasting for more than 60 consecutive seconds were however frequent (51%) and a significant increase in TcPCO_2_ was observed within the 30 minutes following the first drug injection.

The use of atropine before endotracheal intubation is controversial [1,26]. In a recent observational study, 25% of neonates undergoing tracheal intubation in level 3 units experienced bradycardia between 100 and 60 bpm and 21% experienced bradycardia < 60 bpm [27]. In this study atropine use decreased the frequency of bradycardia between 100 and 60 bpm, but not the frequency of bradycradia < 60 bpm. In addition recent results are reassuring regarding atropine use in neonates [28]. Considering these observations and the significance of vagal tone in premature infants [29] we still include atropine as part of our premedication before endotracheal intubation although strong supporting evidence is still lacking.

Opioids have been the most studied drugs for neonatal intubation, either alone or combined to a muscle relaxant [2,4-6,8,9,11,19]. The use of morphine is arguable because of its delayed onset of action [1] and studies showing that it may be less effective than other drug regimens for intubating neonates [5,7]. Rapid onset, short acting opioids used as anaesthetics for tracheal intubation include alfentanil, fentanyl, sufentanil and remifentanil. Sufentanil has shown a faster elimination and a shorter site effect than fentanyl in some circumstances [30]. Thus, it could be the preferred drug in premature infants who usually have altered elimination capacities. Since some recent European studies have reported a frequent use of sufentanil in preterm neonates [16,17] or a trend to increasing its use [18], we consider that data on tolerance and safety are necessary. Remifentanil also seems to be a promising opioid for tracheal intubation either alone [10] or in association with other drugs [5]. Nonetheless, one of its drawbacks is the difficulty to determine its appropriate dose [31].

In 6 other studies associating a synthetic opioid with a muscle relaxant, the first attempt success rate was below 74% (range 35% to 67%) in 4 studies [4,9,11,19] and over 74% (77% and 90%) in 2 studies [2,8]. In a recently published observational study, the overall success rate for the first intubation attempt in neonates across 5 academic level III centres was 44% [32]. For residents, this rate fell to 20.3%. We consider that the rate we observed in our study is rather high, especially among junior operators. The observed success rate of first attempt in a population where 75% of infants had a weight below 1000 g at the time of intubation could be explained, at least partially, by the satisfactory conditions for upper airway visualization as assessed by the operator using the quality of sedation scale. Studies exploring the combination of a fast acting opioid and a muscle relaxant found that experienced personnel had a higher success rate than inexperienced personnel [8,11]. We did not find this difference. This could be explained by a lack of power so we cannot conclude on the efficacy of our regimen on this outcome.

Saturation values ≤ 80% were frequently observed in other studies although usually of shorter duration than in ours [9,11,27]. It should be noted that 99% of infants in Venkatesh et al.’s study [27] and all infants in Lemyre et al.’s study [11] were preoxygenated with 100% FiO_2_. Dempsey et al. [9] recommended to obtain a saturation of at least 95% before starting the procedure. In our study the median FiO_2_ and the median SpO_2_ values one minute before the first drug injection were respectively 37% and 94%. The absence of systematic preoxygenation, of target SpO_2_ recommendation and of positive end-expiratory pressure on our ventilation bags may explain the high incidence of desaturations in our patients. This advocates for proper positive end expiratory pressure use and preoxygenation in very low birth weight infants who are known to have reduced residual functional capacity as compared to older infants [23]. This is probably even more critical when a muscle relaxant is used resulting in additional lung derecruitment. On the other end, oxygen toxicity for the developing eye, brain and lung has been documented [33] and the risk/benefit ratio of preoxygenation on long-term outcome is unknown. Based on our experience, we believe that preoxygenation performed through a face-mask connected to the ventilator circuit should be used so that an effective positive end-expiratory pressure can be provided and peak inspiratory pressure can be controlled; we also believe that a target SpO_2_ value of 95% should be aimed for.

The initial (M5) increase in TcPCO2 we observed is probably due to the laryngoscopy during which no respiratory movement exists due to muscle relaxant use. However we were surprised to observe the persistence of hypercapnia 10, 15 and 30 minutes after the first drug injection. Possible explanations for this may include a major derecruitment at the time of laryngoscopy resulting in collapsed compliance once mechanical ventilation is started or resumed, inappropriate ventilator settings after intubation and/or excessive permissive hypercapnia as illustrated by the similar values observed at the different time points for delta pressure and for respiratory rate. Another possible explanation is a decrease in peripheral perfusion (especially cutaneous) in spite of maintained central perfusion as illustrated by stable blood pressures, resulting in an unreliable transcutaneous PCO_2_ measurement. Unfortunately no arterial blood gas was sampled at that time to confirm or not this hypothesis. Since no other study explored variations in TcPCO_2_ or PCO_2_ during very preterm infants’ intubation we might just have observed a phenomenon that was so far ignored. Future studies should explore gas exchange in the immediate period following intubation.

Thoracic rigidity could possibly be reduced by the injection of the muscle relaxant before the opioid. This sequence can be controversial since paralysis prior to anaesthesia may not be appropriate [34]. However, several publications have reported the injection of a muscle relaxant before an opioid [5,9] based on the hypothesis that obtaining adequate paralysis before the peak opioid effect appears might prevent thoracic rigidity. Therefore, we currently inject atracurium before sufentanil in our unit.

Sedatives such as midazolam or propofol are frequently used as premedications for endotracheal intubation [16,20-22]. Future studies should aim at comparing sedatives with the combination of an opioid and a muscle relaxant. The monitoring of vital signs including desaturations and PCO_2_ should be included in future research.

Our study has several limitations. It is observational and has no comparative group. The collection of data was stopped after 7 months because caregivers were satisfied with this drug combination, this resulted in a limited number of studied cases. We did not record the total number of intubations performed during the study period. Therefore we don’t know if the studied population is representative of all very premature infants intubated in our NICU. Especially we ignore if similar observations would be obtained in emergency intubations. The median postnatal age in our study was 10 days, which is older than the age at intubation observed in other studies [5,10,21,22]. In our unit premature infants born below 28 weeks GA receive prophylactic surfactant in the delivery room. Thus, most intubations performed in very premature infants in our NICU are re-intubations. This explains the age at intubation in our population. Our observations might then not be extrapolated to recently born premature infants. The use of a muscle relaxant precluded the use of any pain scale since body movements or facial expression cannot be evaluated during paralysis. Skin conductance might have provided information on analgesic efficacy of our regimen [35]. However, at the time of our study this technique was not widely in use and the presence of atropine, an anticholinergic drug, in our regimen precluded the use of skin conductance. We did not record time to spontaneous limb and chest movements’ recovery. This information would have helped in the comparison with other opioid plus muscle relaxant regimens. We did not either record the time from decision to intubate to tube fixation which is of interest in an emergency situation. Our definition of the duration of intubation procedure does not provide the time of glottis exposure as others have reported [22]. Interpretation of this parameter is therefore difficult.

## Conclusion

Although the combination of atropine, sufentanil and atracurium for intubating very premature infants offers good intubation conditions without any significant change in patients’ heart rate and blood pressure, proper preoxygenation and alternative drugs to muscle relaxant avoiding paralysis and subsequent lung derecruitment need to be further explored in order to improve tolerance and conditions of neonatal intubation.

## Abbreviations

ETT: Endotracheal tube; GA: Gestational age; IQR: Interquartile range; MAP: Mean arterial blood pressure; NICU: Neonatal intensive care unit; PCO2: Partial pressure of carbon dioxide; RCT: Randomised controlled trial; SpO2: Pulse oxymetry.

## Competing interests

All authors have no conflict of interest concerning this study

## Authors’ contributions

XD designed the study, performed statistical analyses, wrote the first draft, reviewed and revised the manuscript. SD participated to data collection, reviewed and revised the manuscript. PD contributed to statistical analyses, reviewed and revised the manuscript. SB contributed to the study design, reviewed and revised the manuscript. GD contributed to the study design, reviewed and revised the manuscript. LC reviewed and revised the manuscript. RC contributed to study design and statistical analyses, reviewed and revised the manuscript. All authors approved the final manuscript as submitted.

## Pre-publication history

The pre-publication history for this paper can be accessed here:

http://www.biomedcentral.com/1471-2431/14/120/prepub
